# Time-Dependent Effects of Cisplatin on Autophagy Gene Expression in Bladder Cancer Cells

**DOI:** 10.3390/biomedicines14051006

**Published:** 2026-04-28

**Authors:** Sıtkı Ün, Veli Kaan Aydın, Özgür Kurt, Gergana Lengerova, Martina Bozhkova, Steliyan Petrov, Aylin Köseler

**Affiliations:** 1 Department of Urology, Faculty of Medicine, Pamukkale University, Denizli 20160, Turkey; sun@pau.edu.tr; 2 Department of Biophysics, Faculty of Medicine, Pamukkale University, Denizli 20160, Turkey; vkaydin@pau.edu.tr; 3 Department of Medical Microbiology, Acibadem Mehmet Ali Aydinlar University School of Medicine, Istanbul 34752, Turkey; ozgur.kurt@acibadem.edu.tr; 4 Department of Medical Microbiology and Immunology “Prof. Dr. Elissay Yanev”, Medical University of Plovdiv, 4002 Plovdiv, Bulgaria; gergana.lengerova@mu-plovdiv.bg (G.L.); martina.bozhkova@mu-plovdiv.bg (M.B.); steliyan.petrov@mu-plovdiv.bg (S.P.); 5 Center of Competence—Personalized Innovative Medicine, 4002 Plovdiv, Bulgaria; 6 Research Institute, Medical University of Plovdiv, 4002 Plovdiv, Bulgaria

**Keywords:** bladder cancer, cisplatin, autophagy, time-dependent analysis, T24 cells, 5637 cells

## Abstract

**Background/Objectives**: Bladder cancer treatment is frequently hindered by chemoresistance to agents such as cisplatin, a process in which autophagy is hypothesized to play a cytoprotective role. This study aimed to investigate the time-dependent transcriptional dynamics of autophagy-related genes in response to cisplatin in bladder cancer cell lines to better elucidate the molecular underpinnings of this resistance. **Methods**: Two human bladder cancer cell lines, T24 and 5637, were exposed to varying concentrations of cisplatin. Cell viability and half-maximal inhibitory concentration (IC_50_) values were determined at 24 and 48 h using the MTS assay. Subsequently, the relative mRNA expression levels of key autophagy-related genes (*ULK1*, *BECN1*, *ATG5*, *ATG7*, *LC3B*, *SQSTM1/p62*, *LAMP1*, and *TFEB*) were quantitatively analyzed via RT-qPCR at 0, 6, 24, and 48 h intervals. **Results**: Cisplatin exerted a dose- and time-dependent cytotoxic effect, with 5637 cells exhibiting significantly greater sensitivity compared to T24 cells. Transcriptional analysis revealed a dynamic, multiphasic modulation of the autophagic pathway: an early-phase upregulation of initiation genes (*ULK1*, *BECN1*), a mid-phase increase in autophagosome formation genes (*ATG5*, *ATG7*), and a late-phase alteration in lysosomal regulation genes (*LAMP1*, *TFEB*). Notably, the more chemoresistant T24 cells mounted a robust and sustained autophagic transcriptional response, whereas the sensitive 5637 cells demonstrated a more limited and transient reaction. **Conclusions**: Cisplatin modulates the autophagic pathway at the transcriptional level in a highly dynamic, time-dependent, and cell-line-specific manner. Interpreted alongside established functional evidence in the literature, the sustained autophagic gene expression observed in the resistant cells is consistent with a potential cytoprotective role, warranting further functional validation at the protein level. These findings map the temporal genetic landscape of cisplatin-induced autophagy, providing a theoretical framework for optimizing the timing of autophagy-targeted combination therapies in bladder cancer.

## 1. Introduction

Bladder cancer is one of the most prevalent urinary tract malignancies worldwide. Chemotherapy remains one of the main treatment options for this type of cancer [1]. Cisplatin is a cornerstone chemotherapy drug primarily used for bladder cancer treatment. However, the problem with cisplatin is that the patients’ response rate, especially in the metastatic stage, is very low, and most patients develop resistance, which is a major clinical issue [2]. One of the key mechanisms underlying this resistance is the autophagy process, which helps cells survive under stress conditions and can therefore decrease the efficacy of chemotherapy [3]. In this context, studying time-dependent changes in cisplatin-induced autophagy gene expression in bladder cancer cells is of vital importance in revealing the molecular mechanisms of chemoresistance [2]. Autophagy is an evolutionarily conserved lysosomal degradation process that has been demonstrated to play a main role in the adaptation of bladder cancer cells to chemotherapeutic agents and in the development of treatment resistance [4]. A study by Jobst found that autophagy supports cell survival during stress situations, like nutrient deprivation, through the controlled degradation and recycling of cellular components [4]. However, the fact that this process functions as a resistance mechanism to anti-cancer therapies has made autophagy modulation an attractive target for new therapeutic strategies [5]. This resistance mechanism plays a pivotal role, especially in the adaptive response of cells to platinum-based chemotherapeutic agents such as cisplatin [6]. Actually, overcoming the protective autophagy induced by chemotherapy may significantly increase the sensitivity of bladder cancer cells to cisplatin [3]. Notably, functional studies have confirmed that inhibiting this autophagic flux can successfully sensitize these resistant bladder cancer cells to cisplatin-induced death [7].

The activation of the autophagic cascade under chemotherapeutic stress is governed by a complex network of upstream signaling pathways. Cisplatin has been shown to induce autophagy in bladder cancer cells primarily through the activation of the *AMPK* (AMP-activated protein kinase) pathway and the subsequent inhibition of *mTOR* (mammalian target of rapamycin) signaling [8]. This regulatory shift promotes the nuclear translocation of master transcription factors such as TFEB (Transcription Factor EB), which orchestrates the expression of genes involved in both autophagosome formation and lysosomal biogenesis [9].

In this regard, understanding how cisplatin affects these upstream regulators and downstream autophagy pathways in bladder cancer cells is a basis for the development of new treatment approaches targeting resistance mechanisms [10]. In this paper, the time-dependent effects of cisplatin on autophagy gene expression in bladder cancer cell lines are thoroughly studied, thereby providing a comprehensive characterization of the transcriptional autophagic responses associated with chemotherapeutic resistance [11]. This study intends to serve as a reference for future personalized therapeutic approaches by identifying specific genetic mechanisms that play a key role in cisplatin resistance and regulate autophagic flux, which is shown to increase with tumor grade in bladder cancer cells [1,5]. More specifically, a comprehensive analysis of how autophagy gene expression profiles change over time is of great significance to understanding the molecular dynamics of chemoresistance in these cells [12]. Thus, elucidating the dual role of autophagy in supporting cancer cell survival and its complex interactions in tumor development is of vital importance for new cancer treatment strategies [13]. Considering the fundamental function of autophagy in maintaining cellular homeostasis, the dysregulation of this process in cancer cells is closely associated with tumor development, metastasis, and treatment resistance [14,15]. This dual role highlights that autophagy in cancer needs to be modulated very carefully [16]. For instance, autophagy inhibition is common in cisplatin-sensitive cells, whereas in cisplatin-resistant tumor cells, protective autophagy is observed to be highly activated [17]. This indicates that autophagy plays a central role in how cancer cells adapt to chemotherapeutic agents and develop resistance to treatment [17,18]. Therefore, to surpass cisplatin-induced chemoresistance in bladder cancer therapy, understanding the precise molecular chronology of autophagy and targeting these temporal processes are of utmost importance in the development of future therapeutic strategies [18]. While the functional status of autophagy is ultimately determined by protein-level flux, characterizing the temporal transcriptional landscape provides a fundamental blueprint of how cells program their long-term adaptive response to cisplatin stress.

## 2. Materials and Methods

### 2.1. Cell Line and Culture Conditions

Human bladder cancer cell lines T24 and 5637 (ATCC, Manassas, VA, USA) were utilized in this study. Cells were cultured in Dulbecco’s Modified Eagle (DMEM; high glucose; Gibco, Billings, MT, USA) supplemented with 10% fetal bovine serum (FBS; Capricorn Scientific, Ebsdorfergrund, Germany) and 1% penicillin/streptomycin (Gibco, Billings, MT, USA). The cell cultures were maintained in a humidified incubator containing 5% CO_2_ at 37 °C.

### 2.2. Cisplatin Treatment and Cell Viability Assay

Cisplatin (Sigma-Aldrich, St. Louis, MO, USA) was dissolved in 0.9% NaCl and administered to the cells at increasing concentrations. Cell viability was assessed at 24 and 48 h using the MTS assay (Sigma Chemical Co., St. Louis, MO, USA) following exposure to cisplatin concentrations ranging from 0 to 40 µM. Based on the obtained data, dose–response curves were generated, and half-maximal inhibitory concentration (IC_50_) values were calculated for each time point. For subsequent molecular analyses, sublethal doses (close to the IC_50_) that do not cause extreme acute cytotoxicity were selected to allow for the proper investigation of cellular responses.

### 2.3. RNA Isolation and cDNA Synthesis

Total RNA was isolated from the cells at defined time points (0, 6, 24, and 48 h) following cisplatin exposure. The isolation procedure was performed using the Qiagen RNeasy Mini Kit (Qiagen, Hilden, Germany) in accordance with the manufacturer’s instructions. The purity and concentration of the RNA were evaluated using spectrophotometric methods (NanoDrop 2000, Thermo Fisher Scientific, Waltham, MA, USA) and samples meeting the quality criteria (A260/280 ratio ~2.0) were subsequently utilized for complementary DNA (cDNA) synthesis (RevertAid First Strand cDNA Synthesis Kit (Thermo Fisher Scientific, Waltham, MA, USA)).

### 2.4. Real-Time Quantitative PCR (RT-qPCR) Analysis

The expression levels of specific genes representing different stages of the autophagy pathway were quantitatively analyzed using RT-qPCR (CFX96 Real-Time PCR System (Bio-Rad Laboratories, Hercules, CA, USA)). The analyzed gene panel included *ULK1* and *BECN1* (representing the initiation phase), *ATG5*, *ATG7*, and *LC3B* (representing the elongation phase), *SQSTM1/p62* (representing selective autophagy), and *LAMP1* and *TFEB* (representing lysosomal regulation) (Table 1). *GAPDH* was employed as the reference housekeeping gene for normalization. Relative gene expression levels were calculated using the 2^−ΔΔCt^ method to evaluate the time-dependent dynamics of autophagy in response to cisplatin treatment.

### 2.5. Statistical Analysis

All experiments were performed in at least three independent biological replicates. Data are presented as mean ± standard error of the mean (SEM). Statistical analyses and graph generation were conducted using (GraphPad Prism Version 9.0; GraphPad Software, Boston, MA, USA). Comparisons between the control group (0 h) and cisplatin-treated time points (6, 24, and 48 h) were performed using a one-way analysis of variance (ANOVA) followed by Dunnett’s post hoc test. A *p*-value < 0.05 was considered statistically significant.

## 3. Results

### 3.1. Time- and Dose-Dependent Effects of Cisplatin on Cell Viability

**Figure 1:**
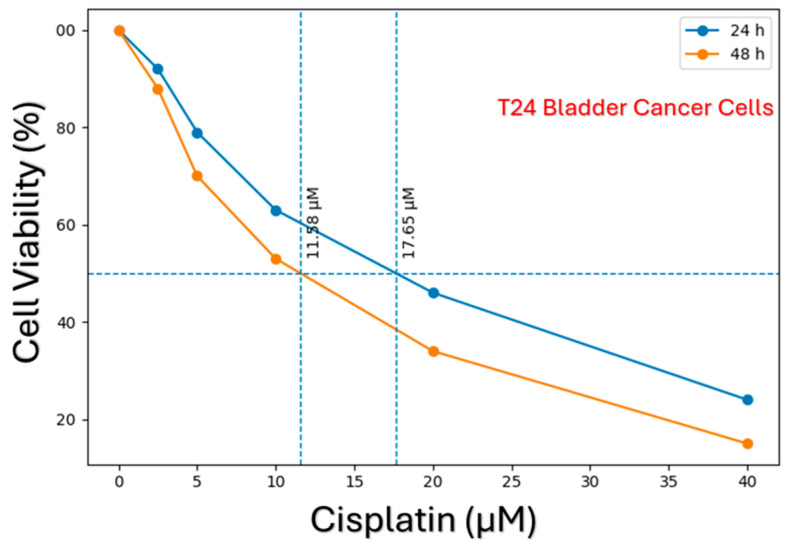
Time-dependent cytotoxic effect of cisplatin and determination of IC_50_ values in T24 bladder cancer cells. Cell viability was evaluated at 24 and 48 h time points following exposure to increasing concentrations of cisplatin (0–40 µM). The resulting dose–response curves demonstrate that cisplatin decreases cell viability in a dose- and time-dependent manner. The horizontal dashed line represents 50% cell viability, while the vertical dashed lines indicate the corresponding IC_50_ values at the respective time points. The lower IC_50_ values observed at 48 h of exposure indicate the increasing cytotoxic effect of cisplatin over time.

The cytotoxic effects of cisplatin on T24 and 5637 bladder cancer cell lines were evaluated in a time- and dose-dependent manner using the MTS assay. The resulting data demonstrated that cisplatin significantly reduced cell viability in both cell lines with increasing concentrations. The dose–response curves obtained for the T24 cell line are presented in Figure 1. In this cell line, a marked decrease in IC_50_ values was observed following 48 h of cisplatin exposure compared to 24 h of exposure. This indicates that the cytotoxic effect of cisplatin increases in a time-dependent manner.

**Figure 2:**
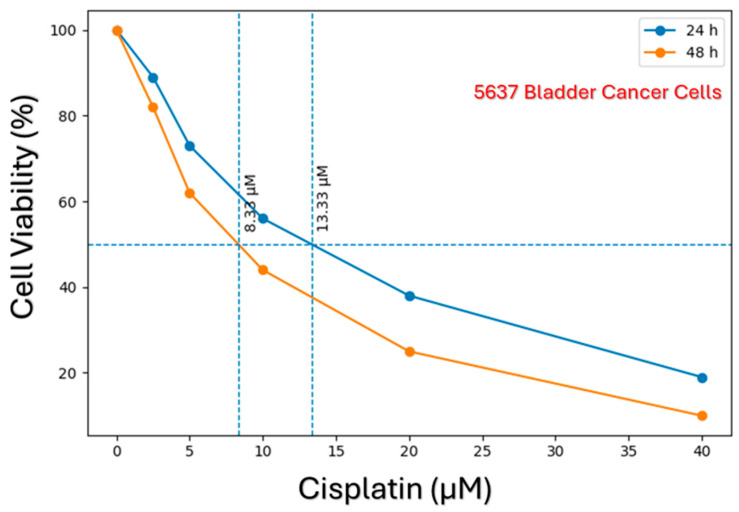
Time-dependent cytotoxic effect of cisplatin and determination of IC_50_ values in 5637 bladder cancer cells. Cells were exposed to varying concentrations of cisplatin for 24 and 48 h, and cell viability was assessed. The dose–response curves indicate a more pronounced sensitivity to cisplatin in 5637 cells. The horizontal dashed line represents 50% cell viability, while the vertical dashed lines indicate the calculated IC_50_ values. Lower IC_50_ values compared to T24 cells demonstrate that this cell line is more sensitive.

Similarly, the cell viability results obtained for the 5637 cell line are shown in Figure 2. A cisplatin-induced decrease in cell viability was also observed in 5637 cells, and the calculated IC_50_ values were notably lower compared to those of the T24 cells. This finding indicates that the 5637 cell line is inherently more sensitive to cisplatin-induced cytotoxicity. Overall, it was demonstrated that while cisplatin exhibits a time- and dose-dependent cytotoxic effect in both cell lines, the magnitude of this effect varies significantly depending on the specific cell line’s resistance profile.

### 3.2. Time-Dependent Changes in Autophagy-Related Gene Expression

**Figure 3:**
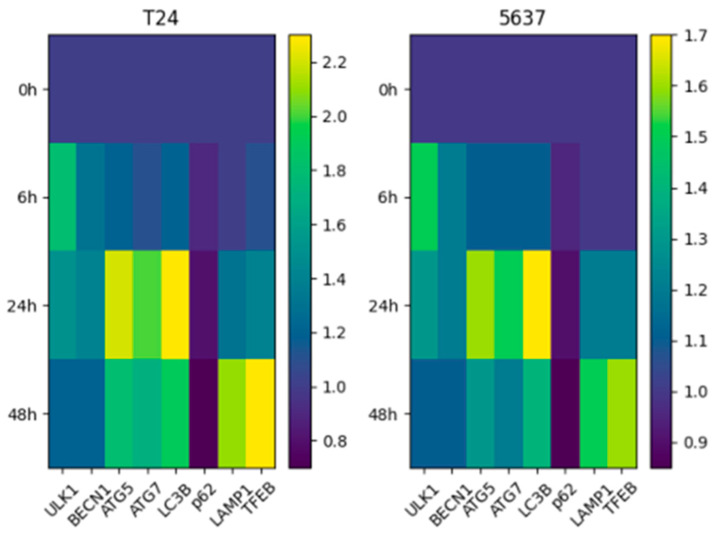
Time-dependent heatmap of autophagy-related gene expression in T24 and 5637 bladder cancer cells following cisplatin treatment. Relative expression levels (2^−ΔΔCt^) are shown for genes involved in autophagy initiation, elongation, selective autophagy, and lysosomal function across 0, 6, 24, and 48 h.

The modulatory effects of cisplatin treatment on autophagy-related gene expression were evaluated in a time-dependent manner. The transcriptional profiles obtained for both cell lines are presented as heatmaps in Figure 3.

This quantitative analysis showed that genes representing distinct stages of the autophagic pathway are dynamically regulated over time. At the early time point (specifically 6 h), an upward trend was observed in the *ULK1* and *BECN1* genes, which govern the initiation stage of autophagy. This suggests that transcriptional program related to autophagy initiation is rapidly induced as a cellular stress response to initial cisplatin exposure. In the middle phase (24 h), the upregulation of *ATG5* and *ATG7* genes, which are responsible for autophagosome formation and elongation, was determined to continue. In contrast, the partial suppression or stabilization observed in certain genes indicates that autophagy is a non-linear and tightly regulated process.

**Figure 4:**
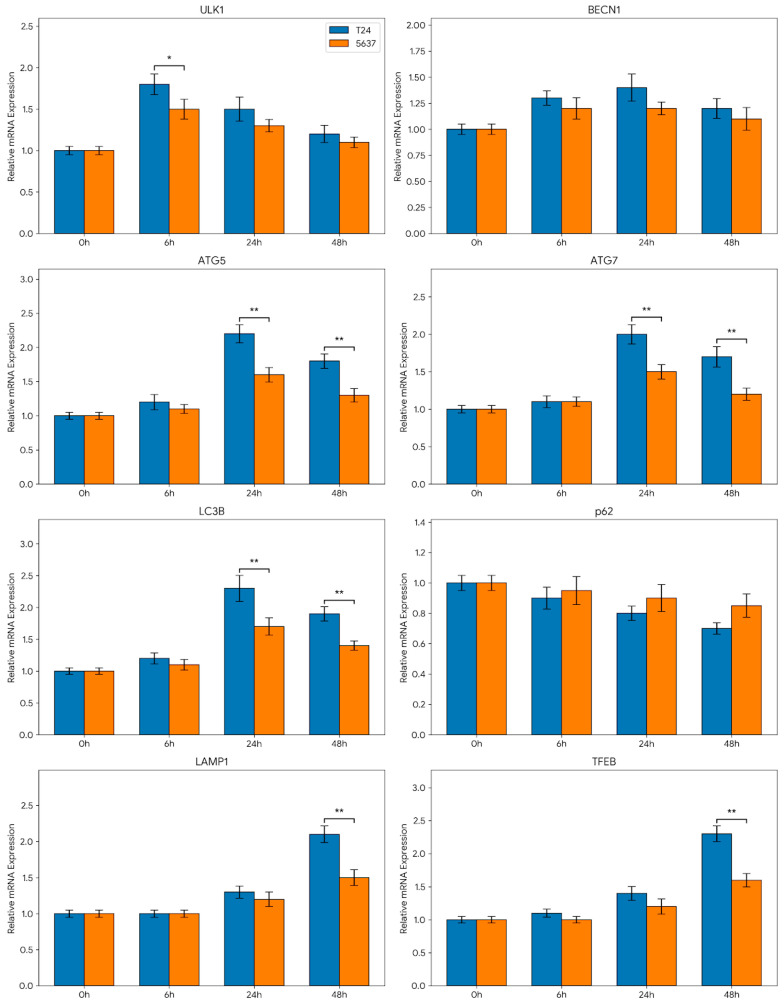
Time-dependent expression profiles of autophagy-related genes in T24 and 5637 bladder cancer cell lines following cisplatin treatment. Relative mRNA expression levels of genes involved in autophagy initiation (*ULK1*, *BECN1*), elongation (*ATG5*, *ATG7*, *LC3B*), selective autophagy (*p62/SQSTM1*), and lysosomal regulation *(LAMP1*, *TFEB*) were evaluated at 0, 6, 24, and 48 h post-treatment. Gene expression levels were calculated using the 2^−ΔΔCt^ method and are presented as fold changes relative to the control group (0 h). Blue bars represent the T24 cell line, and orange bars represent the 5637 cell line. Data are expressed as mean ± SEM of at least three independent experiments. * *p* < 0.05, ** *p* < 0.01 indicating statistical significance between the T24 and 5637 cell lines at the corresponding time points.

In the late phase (48 h), prominent transcriptional alterations were detected, particularly in the *LAMP1* and *TFEB* genes associated with lysosomal regulation. These findings indicate that the autophagy–lysosome axis is heavily involved during prolonged cisplatin exposure, with downstream regulatory mechanisms coming into play in the advanced stages of the cellular response. Furthermore, the expression of the *LC3B* gene, a critical marker of autophagosome formation, was analyzed in a time-dependent manner, and the quantitative results are presented in Figure 4. It was determined that *LC3B* mRNA expression exhibited significant time-dependent changes, revealing cell-line-specific adaptive differences. While a more pronounced and sustainable expression pattern was observed in T24 cells, a more limited and transient transcriptional response was noted in 5637 cells. Overall, the findings reveal that cisplatin modulates the autophagic pathway at the mRNA level in a multi-stage manner, exhibiting distinct dynamics specific to the cell line (Figure 4).

### 3.3. Comparative Analysis Between Cell Lines

When the responses of T24 and 5637 bladder cancer cell lines to cisplatin were evaluated comparatively, prominent phenotypic and molecular differences were observed. According to the cell viability analyses, the 5637 cell line was shown to be highly sensitive to cisplatin, as evidenced by its significantly lower IC_50_ values (Figure 2). Conversely, the higher IC_50_ values of T24 cells demonstrate that this cell line exhibits a more chemoresistant phenotype (Figure 1).

The gene expression analyses strongly parallel this phenotypic divergence. A more distinct and sustainable increase in the expression of autophagy-related genes was observed in T24 cells (Figure 3 and Figure 4). Particularly, the sustained high-level expression of genes involved in the initiation and elongation stages suggests that the autophagic stress response operates as a highly organized and active process in these resistant cells. In contrast, the autophagic transcriptional response in the 5637 cell line was determined to be more limited and transient. Specifically, evaluating the time-dependent changes in *LC3B* and *ATG* genes revealed that the transcriptional drive for autophagy did not maintain continuity in this sensitive cell line, generating a fundamentally weaker adaptive response. This abbreviated response may directly contribute to the vulnerability of 5637 cells to cytotoxic stress.

Furthermore, the more pronounced upregulation of genes associated with lysosomal regulation (especially *TFEB* and *LAMP1*) in T24 cells indicates that the critical autophagy–lysosome axis functions more effectively in this cell line during prolonged stress. It is hypothesized that this molecular mechanism facilitates cellular adaptation to cisplatin, thereby promoting survival. Overall, it can be concluded that the robust and sustainable autophagic transcriptional response observed in the T24 cell line is consistent with a potential cytoprotective role at the transcriptional level, which likely contributes to the development of chemoresistance, whereas the weaker, transient autophagic activity in 5637 cells aligns with their increased sensitivity to the chemotherapeutic agent. The sustained transcriptional drive in T24 cells compared to the transient response in 5637 cells highlights a fundamental difference in how these cell lines program their long-term survival under cisplatin stress. While these transcriptional patterns provide a detailed temporal map of the genetic response to cisplatin, they represent a potential framework that warrants further validation through functional protein-level assays to confirm the complete autophagic flux.

## 4. Discussion

The effects of cisplatin on autophagy-related gene expression in bladder cancer cells were investigated in this research, demonstrating the dynamic, time-dependent, and phase-specific nature of this transcriptional process. The obtained results indicate that autophagy is not a static response but a temporally reprogrammed, multistage adaptive mechanism to cellular stress conditions. Particularly, the autophagic gene expression profiles that are activated in the early phase and differentiated in the late phase in response to cisplatin-induced damage highlight the critical importance of timing for optimizing therapeutic efficacy. In this regard, the increased expression of autophagy-regulatory genes such as *ATG3* and *ATG7* following cisplatin exposure [29], supports activation in the early stages of autophagic flux, while the expression profiles of other genes, such as *ATG10*, *ATG14*, and *LC3B*, may reflect autophagic adaptive responses in later stages [28]. A detailed analysis of these differentiated expression profiles can shed light on specific autophagy mechanisms associated with cisplatin resistance and provide new targets for bladder cancer therapy [30]. For example, it has been reported that increases in *Beclin-1* and *LC3* genes indicate a concentration- and time-dependent induction of autophagy followed by inhibition [31], underscoring the complex interactions of cisplatin with autophagic genes. In addition, the increased expression of major autophagy genes such as *ATG5*, *ATG7*, and *LC3* in cisplatin combination therapies may indicate the potential of synergistic approaches to strengthen the autophagic response [32].

In this context, it is suggested that the transcriptional upregulation of autophagy-positive regulatory genes by agents such as Nectin-4-MMAE in bladder cancer cells could impact the tumor cells’ response to treatment and potentially offer new strategies for overcoming chemotherapeutic resistance [33]. These results provide evidence that the induction of autophagy likely leads to the enhancement of the cytotoxic effects of agents like Nectin-4-MMAE, thereby creating new therapeutic avenues in bladder cancer treatment [33]. Within this framework, alterations in the expression patterns of genes regulating autophagy, particularly the modulation of key effectors such as *ATG5*, *ATG7*, and *LC3*, may offer potential biomarkers and therapeutic targets for overcoming chemoresistance [2,16,33]. The increased expression of these genes in response to cisplatin treatment is consistent with a putative cytoprotective role of autophagy within these cancer cells [15]. Consequently, in scenarios where autophagy supports cancer cell survival, the combination therapy of autophagy inhibitors with cisplatin might result in enhanced chemotherapeutic efficacy [15].

Our current findings demonstrate that during the early phase (specifically around 6 h), there is a distinct upregulation in genes operating at the autophagy initiation steps (especially *ULK1*), implying the rapid activation of a cellular stress response against cisplatin. This early activation suggests that cells under cytotoxic stress prime the autophagic transcriptional program, potentially as a protective survival mechanism. In fact, early autophagic responses to chemotherapeutic agents have been shown to play a vital role in maintaining cellular homeostasis. For instance, in dichloroacetate combined cisplatin treatment, the expression of autophagy-related genes increases, supporting the premise that autophagy acts as a protective buffer against chemotherapeutic stress [32]. Thus, cisplatin-induced autophagy has the potential to increase chemoresistance in bladder cancer cells, making the targeted modulation of this pathway highly significant for future therapies [2]. Therefore, studying the role of autophagy in cancer development and investigating microRNAs related to dysregulated autophagy may aid in the development of promising therapeutic strategies [34].

During the middle phase (24 h), the upregulation of genes such as *ATG5* and *ATG7*—which are crucial for the formation and elongation of the autophagosome—points to the progression of the autophagic process and its structurally active state. This suggests that the transcriptional drive for autophagy is not only initiated but maintained, supporting the active recycling of intracellular components. Conversely, the partial repression or stabilization of certain genes at this point implies that autophagy is not a simple linear increase but rather a tightly regulated cascade. This highlights the critical balancing role autophagy plays in cancer cell survival mechanisms, a balance that can be manipulated through strategic therapeutic interventions [11]. In the delayed phase, the reduction in specific autophagy genes raises the possibility that prolonged exposure to cisplatin might eventually disrupt autophagic flux or inhibit the process, thereby pushing the cell toward alternative death pathways, such as apoptosis. This reinforces the dynamic role of autophagy in cellular adaptation and suggests that modulating the autophagic response during long-term exposure could significantly enhance chemotherapy efficacy [29].

Late-phase (48 h) changes, particularly those related to lysosomal function and autophagy–lysosomal axis genes (*LAMP1*, *TFEB*), were highly significant. This finding indicates that during long-term cisplatin exposure, the transcriptional response is not limited to autophagosome formation; rather, lysosomal activity is heavily integrated into the adaptive process. Notably, the increased expression of *TFEB* suggests that lysosome biogenesis and autophagic flux are regulated in a highly coordinated manner. Consequently, the autophagy–lysosome axis emerges as a major component of cellular adaptation in the late phase, potentially driving the development of chemoresistance [12]. Hence, characterizing this dynamic progression could facilitate the development of promising treatment strategies [34]. As a master transcription factor regulating the expression of lysosomal biogenesis and autophagy genes, *TFEB* is highly likely to play a pivotal role in modulating this adaptive response [35,36,37]. In this regard, it has been reported that *TFEB* activation is associated with cisplatin resistance and may serve as a prognostic marker for chemoresistance in various malignancies, including ovarian cancer [38]. *TFEB* has been shown to stimulate doxorubicin resistance by promoting survival in colon and cervical cancer cells, while in glioblastoma, it alters cellular metabolism and reduces chemosensitivity [39]. Therefore, manipulating *TFEB* activity in bladder cancer cells by modulating the cisplatin-induced autophagic response holds considerable potential for enhancing therapeutic efficacy [38]. The dual role of *TFEB* in endosomal maturation and autophagy regulation is partially explained by its control over PtdIns3P levels, which affects lysosome positioning [40]. Thus, the complex interaction of *TFEB* between the endosomal system and autophagy presents a critical target for comprehensively understanding the bladder cancer cell response to cisplatin [40].

Comparative analysis of the cell lines revealed that 5637 cells are inherently more sensitive to cisplatin than T24 cells, as supported by their notably lower IC50 values. Concurrently, the autophagic transcriptional response in T24 cells was observed to be far more organized and sustainable, with lysosomal integration becoming particularly pronounced in the late phase. This molecular divergence suggests a correlation between a robust autophagic transcriptional response and the chemoresistant phenotype observed in specific cell lines. Conversely, in the more sensitive 5637 cells, the autophagic process lacked continuity, and lysosomal integration appeared insufficient, rendering these cells substantially more vulnerable to cytotoxic stress. This highlights the absolute necessity of developing personalized approaches in bladder cancer treatment and underscores the need for an in-depth investigation into how autophagy modulates chemotherapeutic responses. Given *TFEB*’s central role in regulating autophagy–lysosome biogenesis and metabolism [41,42,43], a detailed elucidation of cisplatin-induced *TFEB* activation mechanisms across different cell lines is vital for assessing its potential as a predictive biomarker [42,43,44]. Detailed analysis of *TFEB* nuclear translocation and its paracrine/autocrine regulation could yield novel strategies to overcome chemoresistance [45,46]. Furthermore, by controlling PtdIns3P levels, *TFEB* optimizes lysosomal degradation capacity, emerging as a key resistance mechanism [40]. Further molecular elucidation could provide a robust foundation for *TFEB*-targeted therapeutic agents.

These results support the well-documented notion that autophagy plays a paradoxical, dual role in cancer cells (acting as both a protective and cell-death-promoting mechanism) [47,48]. Often described as a ‘double-edged sword,’ autophagy can act as a tumor suppressor in early stages but transition into a pro-tumoral mechanism that manages metabolic stress in advanced tumors, thereby increasing chemoresistance [47,49,50]. Performing time-dependent analyses provides critical clues regarding the precise temporal phases at which autophagy should be therapeutically targeted. Early phase inhibition might reduce the cells’ ability to manage stress and lower their ‘metabolic fitness’ [48,51]; conversely, targeting lysosomal functions (e.g., with chloroquine-like agents) during the late phase can disrupt autophagic flux, leading to the lethal accumulation of cytotoxic products [52,53,54]. Understanding these kinetics and optimizing treatment protocols according to these temporal windows is emerging as a critical clinical approach for breaking chemotherapeutic resistance [55,56].

### Study Limitations and Contextual Validation

While our study provides a comprehensive temporal map of autophagic gene expression, we acknowledge several critical limitations. Foremost, this study relies primarily on mRNA expression data obtained via RT-qPCR. Since transcriptional changes do not always perfectly correlate with functional protein activity or enzyme activation due to post-translational modifications [57]. the lack of Western blot validation (e.g., LC3-I to LC3-II conversion, p62 degradation) and functional autophagic flux assays (e.g., utilizing mRFP-GFP-LC3 reporters or lysosomal inhibitors like chloroquine or bafilomycin A1) limits the direct confirmation of actual autophagic executions [57,58,59,60,61]. It has been noted in the literature that chemotherapeutic agents may increase cytotoxic damage by blocking autophagic flow at the lysosomal stage rather than inducing true autophagic flux [53,55]. Furthermore, while we documented the upregulation of *TFEB*, we did not experimentally assess upstream regulatory events, such as *mTOR/AMPK* signaling alterations or *TFEB* nuclear translocation.

Nevertheless, our temporal transcriptional data robustly aligns with established functional models in the literature. Independent studies have previously demonstrated through Western blotting and Transmission Electron Microscopy (TEM) that cisplatin induces genuine autophagic flux in T24 and 5637 cell lines, characterized by significant LC3-II accumulation and p62 clearance [7]. Furthermore, the upstream regulation of this process via the *AMPK/mTOR* axis has been functionally mapped in bladder cancer models [8,9]. Our findings provide the vital temporal transcriptional blueprint that precedes and sustains these documented protein-level changes. Finally, we proposed a cytoprotective role for autophagy in the resistant T24 cell line. Although we did not perform functional rescue experiments or flow cytometry-based apoptosis assays combining cisplatin with autophagy inhibitors in this specific study, the functional causality of this mechanism is well-supported by external evidence. Data from established literature demonstrate that pharmacological inhibition (via chloroquine) or genetic silencing of essential autophagy genes (such as *ATG7* or *BECN1*) significantly sensitize these specific bladder cancer cells to cisplatin-induced apoptosis [7]. Therefore, our temporal gene expression profiling accurately reflects the underlying genetic reprogramming that drives this established cytoprotective phenotype.

## 5. Conclusions

This study elucidates the time-dependent effects of cisplatin treatment on autophagy-related gene expression profiles in bladder cancer cell lines (T24 and 5637), exploring the molecular chronology of chemotherapeutic resistance. The data generated demonstrate that cisplatin-induced cellular stress triggers the transcriptional activation of key autophagy genes such as *ULK1*, *BECN1*, *ATG5*, and *ATG7*; however, the intensity and sustainability of this response depend heavily on the inherent chemoresistance profile of the cell line [62]. Specifically, in the phenotypically resistant T24 cells, the continually elevated expression of genes associated with the autophagic initiation and elongation complexes is perfectly in line with the “protective autophagy” concept [2,10]. It is highly probable that T24 cells preserve their metabolic homeostasis by employing this autophagic transcriptional program as a survival strategy against cisplatin, making this process a critical step in chemoresistance [2,4]. In contrast, the limited and transient autophagic response in 5637 cells implies that these cells remain much more vulnerable to cisplatin-induced DNA damage and oxidative stress [10,11].

Our transcriptional data, interpreted within the context of existing functional literature, supports the dual role of autophagy in cancer therapy [14,47]. From a clinical perspective, the silencing of genes such as *Beclin-1*, *ATG5*, and *ATG12* has been demonstrated to sensitize bladder cancer cells to cisplatin [3,5]. In this sense, the robust genetic upregulation observed in T24 cells provides a strong theoretical basis supporting the therapeutic potential of autophagy inhibitors in combination with cisplatin, especially in chemoresistant cases [2,15]. Moreover, the marked late-phase changes in *TFEB* and *LAMP1* further highlight lysosomal biogenesis as an integral part of chemotherapeutic adaptation [12,40]. Combining these detailed temporal genetic blueprints with functional autophagic flux analyses in future targeted studies will lay the groundwork for the development of more potent, precisely timed, and personalized treatment strategies to overcome chemoresistance in bladder cancer, provided that these transcriptional profiles are further validated in clinical settings [5,15].

## Figures and Tables

**Table 1:** Primer sequences for RT-qPCR analysis.

Gene	Forward Primer (5′–3′)	Reverse Primer (5′–3′)	Reference
*ULK1*	AGCAGTTGCTTCTGTTGCTC	TCTGCTGATGGTGATGTTGG	[19]
*TFEB*	CAGTGGAGCAGAGAGACTTTG	CTCTTCTGGTAGCTGCTGGT	[20]
*SQSTM1*	AGGCGCACTACCGCGAT	CGTCACTGGAAAAGGCAACC	[21]
*NBR1*	TGGACAGCTTCAGTCTTCGT	CTGTAGGTGGAGGCTTTGGT	[22]
*ATG5*	CAGTTTGGCACACACTTGTG	GTCTGTGATGGGTTGTTGCT	[23]
*ATG7*	ACACCAAGAGGAGCTGTTGA	TGTGCTGTTGCTGTAGGTGT	[24]
*LC3B*	GAGAAGCAGCTTCCTGTTCT	CTCCTGGGAGGCATAGACAT	[25]
*BECN1*	GGGGATCCGGAAGTTTTCGGCGGCTA	GGGGGAATTCGAAGAAAGGGAAAGGAGT	[26]
*GAPDH*	GAAGGTGAAGGTCGGAGTC	GAAGATGGTGATGGGATTTC	[27]
*LAMP1*	ACGTTACAGCGTCCAGCTCA	TCTTTGGAGCTCGCATTGG	[28]

## Data Availability

The original contributions presented in this study are included in the article. Further inquiries can be directed to the corresponding author.

## Abbreviations

The following abbreviations are used in this manuscript:

ATG	Autophagy-related gene
BECN1	Beclin 1
cDNA	Complementary DNA
DCA	Dichloroacetate
DMEM	Dulbecco’s Modified Eagle Medium
FBS	Fetal bovine serum
GAPDH	Glyceraldehyde 3-phosphate dehydrogenase
IC50	Half-maximal inhibitory concentration
LAMP1	Lysosomal-associated membrane protein 1
LC3/LC3B	Microtubule-associated protein 1 light chain 3
MMAE	Monomethyl auristatin E
mRFP-GFP	Monomeric red fluorescent protein-green fluorescent protein
MTS	3-(4,5-dimethylthiazol-2-yl)-5-(3-carboxymethoxyphenyl)-2-(4-sulfophenyl)-2H-tetrazolium
PtdIns3P	Phosphatidylinositol-3-phosphate
RT-qPCR	Reverse transcription quantitative polymerase chain reaction
SQSTM1/p62	Sequestosome 1
TFEB	Transcription factor EB
ULK1	Unc-51-like autophagy-activating kinase 1
